# An Overview of the Measurement of Permeability of Composite Reinforcements

**DOI:** 10.3390/polym15030728

**Published:** 2023-01-31

**Authors:** Andrea Dei Sommi, Francesca Lionetto, Alfonso Maffezzoli

**Affiliations:** Department of Engineering for Innovation, University of Salento, Via Arnesano, 73100 Lecce, Italy

**Keywords:** permeability measurement, Darcy’s law, resin flow, flow front monitoring, composite materials, liquid composite molding

## Abstract

Liquid composite molding (LCM) is a class of fast and cheap processes suitable for the fabrication of large parts with good geometrical and mechanical properties. One of the main steps in an LCM process is represented by the filling stage, during which a reinforcing fiber preform is impregnated with a low-viscosity resin. Darcy’s permeability is the key property for the filling stage, not usually available and depending on several factors. Permeability is also essential in computational modeling to reduce costly trial-and-error procedures during composite manufacturing. This review aims to present the most used and recent methods for permeability measurement. Several solutions, introduced to monitor resin flow within the preform and to calculate the in-plane and out-of-plane permeability, will be presented. Finally, the new trends toward reliable methods based mainly on non-invasive and possibly integrated sensors will be described.

## 1. Introduction

Liquid composite molding (LCM) processes are composite manufacturing techniques in which a dry fibrous reinforcement is impregnated by a liquid resin inside a sealed cavity. The driving force for the impregnation is the pressure difference between a resin inlet and an outlet vent [1,2,3]. Several processes belong to the family of LCM. Resin Transfer Molding (RTM), for instance, exploits positive pressures and requires rigid mold halves, while in Vacuum Assisted Resin Infusion (VARI), a vacuum is applied, and a vacuum bag is used in place of the upper part of the mold. For more details on the technologies, the readers are referred to recent literature [4,5]. In particular, VARI has several advantages allowing the fabrication of large parts with high performance and complex geometry at a lower cost compared to other technologies and with a lower content of voids and volatile organic compound emission compared to hand lay-up. However, the diffusion of LCM technologies is still limited by reproducibility, related to some issues such as fiber displacement, preform deformation, and high fiber volume fractions hardly achievable [6].

One of the most critical properties which strongly affect processing time and porosities in the final composite part is the reinforcement permeability, which depends both on reinforcement material, preform architecture, resin properties, and process parameters. In addition, permeability is an anisotropic property, and consequently, its value may significantly differ depending on the directions of the resin flow. For these reasons, the permeability value is not provided by reinforcement manufacturers but must be determined for each specific case.

Despite several benchmark exercises for the measurement of in-plane [7,8,9] and out-of-plane [10] permeability and suggestions proposed for permeability measurement experiments [11,12], test standardization has not been achieved yet. This is due to several experimental difficulties related to material properties, intrinsic limitations of experimental procedures, and human errors.

Computational fluid dynamics (CFD) analysis can be a useful tool to optimize the fabrication process of components with complex geometry and high-performance requirements, thus, reducing costly trial-and-error approaches. Several pieces of software are suitable for simulating an LCM process, and, among others, Moldex3D (CoreTech System Co., Zhubei, Taiwan) [13], PAM-RTM (ESI Group, Rungis, France) [14], Moldflow (Autodesk, San Francisco, California, USA) [15], and RTM-Worx (Polyworx, Nijverdal, The Netherlands) [16]. In addition, general-purpose computational software such as COMSOL Multiphysics (COMSOL, Burlington, Massachusetts, USA) [17], ANSYS Fluent (ANSYS, Inc., Canonsburg, Pennsylvania, USA) [18], and the open-source OpenFOAM (OpenFOAM Foundation, London, England, UK) [19] can be used. Generally, the commercial software developed for the filling simulation in an RTM or VARI process, such as PAM-RTM and RTM-Worx, exploit a macro-scale approach based on Darcy’s law, while the general-purpose fluid dynamics packages ANSYS Fluent and OpenFOAM solve conservation equations for momentum and mass [20,21]. Yang et al. [22], for instance, proved the effectiveness of CFD analysis by simulating the saturated through-thickness flow to optimize the mold design used for the permeability experiments. They performed steady-state laminar flow simulations with the software ANSYS Fluent. This software was also applied to predict permeability from 3D X-ray microtomography images [23,24].

**Figure 1:**
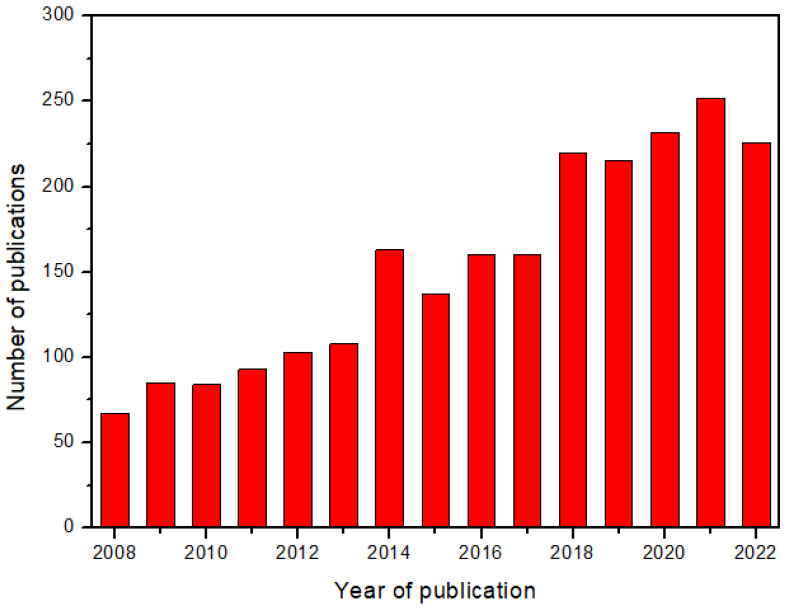
Trends in scientific publications focused on reinforcement permeability in the last fifteen years (2008–2022) [25].

However, CFD analysis requires knowledge of the permeability values. For this reason, a lot of research studies have been carried out in the last fifteen years, focusing on the measurement of reinforcement permeability. The interest in this topic has continuously increased, as demonstrated by the trend in the number of scientific publications reported in Figure 1.

Relevant reviews available in the literature include the work of Naik et al. (2014) [26] concerning the effects of resin and reinforcement properties and process parameters on the permeability of polymer matrix composites, and that of Pantaloni et al. (2020) [27], on flow simulation and permeability measurement for plant fiber reinforcements considering the effects of characteristic phenomena such as fluid absorption and preform swelling. In addition, Michaud (2016) [28] focused on the non-saturated resin flow in LCM processes. Karaki et al. (2019) [29] showed progress in experimental and theoretical methods for permeability evaluation but without a detailed description of the experimental procedure. However, after the work of Sharma et al., in 2010 [30], a specific review focused on the experimental methods for the permeability measurement in fiber-reinforced composites is missing, although the number of papers published on this subject is rather large, with an average of about 230 papers per years in the last five years. Therefore, this work aims to realize an updated overview of the permeability measurement methods focused on the advancements in the last six years. The description of the principles underlying each measurement method and of the adopted experimental set-up will be addressed, emphasizing the strength and drawbacks of each method.

## 2. Permeability

The impregnation of the dry preform with liquid resin can be modeled as a flow through a porous medium, whose geometrical features are characterized by its permeability. According to this approach, the fluid superficial velocity $\vec{v}$ (m/s) can be deduced from Darcy’s law [31]:

(1) $$\vec{v}=-\frac{K}{\eta }\nabla P$$

where η (Pa × s) is the resin viscosity, K (m^2^) is the permeability tensor of the preform, and ∇P (Pa/m) is the pressure gradient. Permeability, in the general case of an anisotropic material, is a second-order tensor of the following type:

(2) $$K=\left(\begin{array}{ccc} K_{\mathrm{xx}} & K_{\mathrm{xy}} & K_{\mathrm{xz}}\\ K_{\mathrm{yx}} & K_{\mathrm{yy}} & K_{\mathrm{yz}}\\ K_{\mathrm{zx}} & K_{\mathrm{zy}} & K_{\mathrm{zz}} \end{array}\right)$$

**Figure 2:**
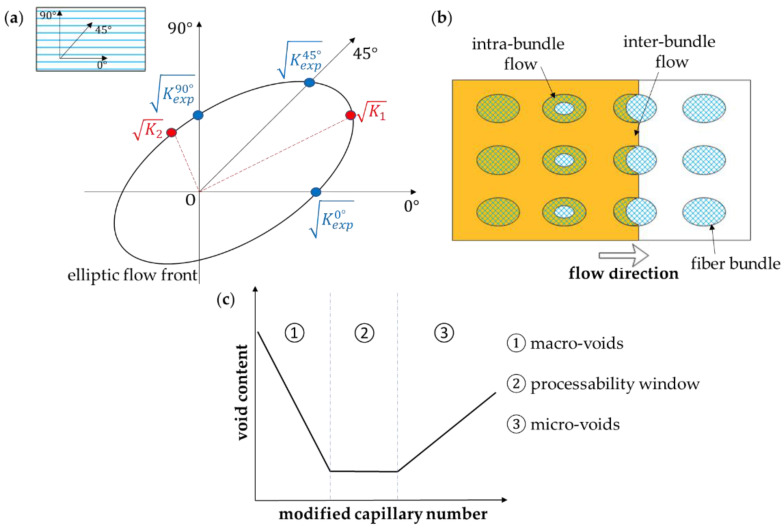
(**a**) In-plane elliptic flowing pattern with the square roots of K_1_ and K_2_ as the major and minor semi-axis, respectively ([33] Copyright (2007) with permission from John Wiley and Sons), (**b**) illustration of the dual-scale flow ([35] Copyright (2006) with permission from Elsevier), and (**c**) a typical plot of the void content versus the modified capillary number ([36] Copyright (2006) with permission from Elsevier).

If the principal permeability directions are considered, this tensor can be diagonalized:

(3) $$K=\left(\begin{array}{ccc} K_{1} & 0 & 0\\ 0 & K_{2} & 0\\ 0 & 0 & K_{3} \end{array}\right)$$

where K_1_ and K_2_ are the in-plane permeabilities (in-plane refers to the textile layer) and K_3_ is the out-of-plane permeability [9]. Weitzenböch et al. [32] developed a method to determine the principal permeabilities K_1_, K_2_, and K_3_ through six unidirectional flow experiments conducted along different directions. If only in-plane permeabilities K_1_ and K_2_ are considered, three experiments along directions with an offset of 45° are sufficient (Figure 2a). Demaría et al. [33] introduced the iterative ellipse method valid for the general case of arbitrary orientations. Di Fratta et al. [34] reduced the number of the required unidirectional experiments to two or even one by considering the angle that the flow front forms with the measurement direction.

Actually, resin flow consists of two different flows, an inter-bundle meso-flow, driven by the applied pressure gradient, and an intra-bundle micro-flow, driven by capillary pressure (Figure 2b). This is important because if micro-flow is dominant, inter-bundle mesoscale voids are formed, while inter-bundle micro-scale voids are expected when meso-flow predominates. Labat et al. [37] defined a “processability window” representing the range of injection parameters leading to a minimum void content (Figure 2c). Ruiz et al. [38], moreover, showed how to reduce the formation of macro- and micro-voids by correcting the injection flow rate or pressure at each time step, thus, ensuring an optimal value of a modified capillary number corresponding to the ratio of the capillary number to the liquid/fiber contact angle [39], at the fluid flow front position. A high modified capillary number usually occurs in industrial LCM processes as a consequence of high applied pressures (of the order of 0.1 MPa or higher) and fluid velocity. So as shown in Figure 2c, micro-void defects are expected. Although it is quite difficult to evaluate this complex dual-scale flow, several works have been conducted to study this phenomenon [40,41,42,43,44,45]. In the macro-scale approach, the global flow is considered with an average value of fluid velocity and permeability.

Capillary pressures are responsible for the difference in unsaturated and saturated permeability, where saturated permeability refers to a fully impregnated preform and depends on many factors, such as fiber geometry, orientation, and wettability [26]. In the saturated case, resin flow encounters less resistance compared to dry preform since there is a uniform saturated flow. As a consequence, saturated permeability values are generally higher than unsaturated permeability ones [11]. Some authors [46,47,48] introduced the relative permeability to take into account the effect of the degree of saturation on the flow, defined as the ratio of liquid to pore volume. Moreover, it is important to consider the effects of the presence of binders on the preform permeability [49] and the potential viscosity change during infusion. For that reason, during a permeability test, it is recommended to also measure the viscosity of the fluid which exits the mold [12].

## 3. Measurement Methods

Many different methods have been proposed to measure permeability. They can be classified according to the flow direction (linear or radial), flow state (transient/unsaturated or steady state/saturated), and injection conditions (constant flow rate or constant injection pressure). In addition, if a sensor is adopted, it can be divided into point, lineal, and areal sensing methods. In this section, methods are presented by considering the most popular techniques adopted in the last six years. The analyzed experimental methods are resumed in Table 1 with their main advantages and drawbacks. Each method has proved to be valid in terms of accuracy, taking into account the inherent sensitivity of the equipment used. The benchmark exercises demonstrated the reproducibility of measurements as long as the test conditions were properly controlled.

### 3.1. Methods Based on Flow Rate Measurement

One relatively simple method to determine in-plane and out-of-plane saturated permeability K_sat_ is based on the measurement of the volumetric flow rate, from which K_sat_ can be obtained by using the following formulation of Darcy’s law:

(4) $$Q=-\frac{\mathrm{KsatA}}{\eta }\frac{\Delta P}{L}$$

where Q (m^3^/s) is the volumetric flow rate, A (m^2^) is the flow channel cross-sectional area, ΔP (Pa) is the pressure difference between the fluid inlet and outlet, η (Pa × s) the fluid viscosity, and L (m) the specimen thickness, respectively.

This method is generally used for determining out-of-plane saturated permeability K_3-sat_ [10] and does not require particular competence or instrumentation except for a flow meter and a pressure gauge. Aziz et al. [50] determined the transverse permeability of dry carbon fiber preforms manufactured by automated fiber placement with this method. Rimmel et al. [51] assessed the beneficial impact of stitching on the out-of-plane permeability of carbon fiber preforms manufactured by fiber placement. In particular, stitching induced a higher permeability by about a factor of 50 with a smaller standard deviation due to the presence of flow channels in the microstructure of the stitched preforms.

**Figure 3:**
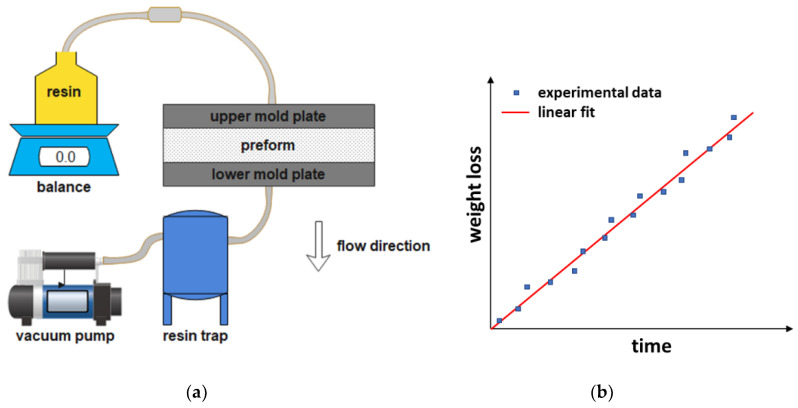
(**a**) Experimental set-up for gravimetric method and (**b**) a typical plot of the weight loss versus time (adapted from Lionetto et al. [52]).

As an alternative to measuring the volumetric permeability, the mass permeability can be measured. Mass flow rate can be obtained from weight measurements, as shown by Lionetto et al. [52], who determined the permeability of three different carbon fiber preforms, made of a balanced fabric, a stitched unidirectional fabric, and a unidirectional one, at different values of fiber volume fraction V_f_. Polyethylene glycol 400 (PEG400) was used as a test fluid. The experimental set-up shown in Figure 3a consists of a vessel containing the test fluid positioned on a balance, the mold, and a vacuum pump, to realize a VARI process under constant flow rate and pressure. When the preform is saturated, and all air has been displaced from the flow channels, the steady state condition is achieved, and the quantity of fluid that passes through the thickness of the preform can be measured by the balance.

Out-of-plane permeability K_3-sat_ is obtained from the slope of the plot of the fluid weight loss as a function of time t (s) by the following equation:

(5) $$\mathrm{QAt}=\mathrm{Weight}=\frac{{\rho K}_{3-\mathrm{sat}}A}{\eta }\frac{\Delta P}{L}t$$

where ρ (kg/m^3^) is the fluid density, which is used for calculating the volumetric flow rate. A typical plot of the weight loss versus time is shown in Figure 3b. In all cases, permeability decreased as the fiber volume fraction increased, and the balanced preform showed the highest permeability values. Between the two unidirectional preforms, permeability was higher for the stitched one.

Using the gravimetric method, Kabachi et al. [53] studied the effects of cyclic reinforcement compaction on out-of-plane saturated permeability. The permeability of three glass fiber reinforcements, a woven fabric, a non-crimp fabric, and a mat, was determined before and after 100 compaction cycles up to a thickness of 4 mm with a rate of 24 mm/min at different values of fiber volume fraction. Each circular ply sample of 119 mm diameter was placed between two steel plates and impregnated with silicone oil, while pressure drop and flow rate were monitored using two pressure sensors and a balance, respectively. Permeability decreased with increased fiber volume fraction, while the cyclic compaction increased the textile nesting into a denser configuration but did not affect the permeability values, reducing, however, the standard deviation in the results. Caglar et al. [54], instead, applied the gravimetric method to study the effects of spherical inclusions on the in-plane saturated permeability K_1-sat_ of a 2 × 2 twill weave glass fabric. Permeability decreased for low bead contents and diameters, while it increased with larger beads, which formed new pores or enlarged existing ones.

**Figure 4:**
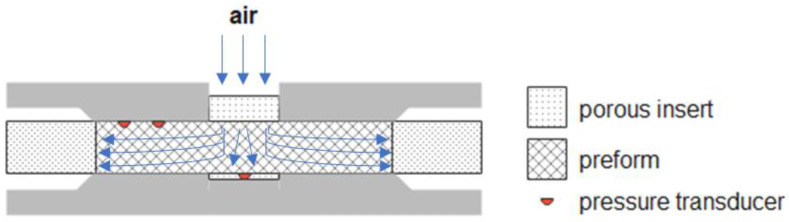
Schematic cross-section of experimental set-up for air flow measurement ([56], Copyright (2022) with permission from Elsevier).

Samples tested for permeability measurement are generally disposed of after the experiment. Hermann et al. [55] designed a prototype to perform non-destructive measurements of flow resistance by monitoring the decay with the time of an air pressure pulse for in-plane and through-thickness configurations. A good correlation between injectability and permeability was verified. Abdoli et al. [56] proposed a novel non-destructive technique based on air as the test fluid. As sketched in Figure 4, each sample of eight different reinforcements was placed between two circular platens (200 mm diameter), and the air was injected from the top. Air pressures and flow rate were measured by pressure transducers and flow meter, respectively, and the collected data were processed by artificial intelligence, a trained Neural Network, to predict the full permeability tensor. The results were not fully satisfactory, and further work is needed to improve the Neural Network predictions.

### 3.2. Methods Based on Video Recording

**Figure 5:**
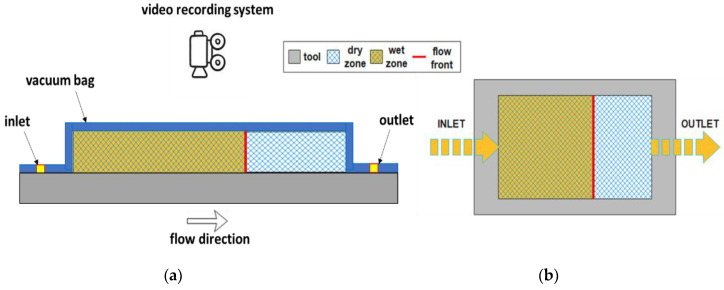
(**a**) Experimental set-up for video recording method and (**b**) a sketch of in-plane flow front advancement.

Visual monitoring of the flow front position is generally used to determine in-plane unsaturated permeability and is one of the most used methods, as it requires only a transparent mold and a video camera to record the flow front evolution during infusion. The injection can be either linear or radial. Lionetto et al. [57] calculated the in-plane permeability of two types of unidirectional carbon fiber fabrics with polyethylene glycol 400 (PEG400) as a test fluid. The experimental set-up is sketched in Figure 5a. The transparent vacuum bag, used for the resin infusion process, enables one to visualize the flow front during infusion, recorded by a video camera, as shown in Figure 5b. A flat flow front must be observed in the absence of low permeability fluid bypass paths, which can alter the measurement.

In-plane permeability is obtained by integrating Darcy’s equation for one-directional flow under a constant pressure, where the linear velocity of the fluid is considered [58]:

(6) $$x_{f}=\sqrt{\frac{2K\Delta P}{{\eta (1-V}_{f})}}\sqrt{t}$$

where x_f_ (m) is the flow front position recorded at each time t (s), η (Pa × s) is the fluid viscosity, V_f_ (-) is the fiber volume fraction, and ΔP (Pa) is the pressure drop. By extrapolating the slope of the linear fitting of the experimental data, it is possible to calculate the permeability K along the flow direction:

(7) $$K=\frac{{\left(\mathrm{slope}\right)}^{2}\cdot {\eta (1-V}_{f})}{2\Delta P}$$

This method enables us to obtain the two values of in-plane permeability, K_1_ or K_2_, from two distinct experiments where the preform is oriented at 0° and 90° with respect to the flow direction. Compared to the unstitched unidirectional preform, the stitched one showed comparable permeability values along the two directions.

Da Silva et al. [59] applied this method to investigate the effect of hybridization on the in-plane permeability of R-glass/aramid/epoxy composites, proving that the inclusion of R-glass layers determines an increased permeability compared to pure aramid composite. De Oliveira et al. [60] evaluated the in-plane permeability of coconut fiber mats with and without-an atmospheric plasma treatment. The test was performed with a solution of non-reactive glycerin, water, and colorant as a test fluid. The treated mat showed a lower permeability value but a more homogeneous impregnation without entrapped air. When natural fibers are used with water solution or hydrophilic fluids, the effect of phenomena such as capillarity, liquid absorption, and preform swelling on permeability must be considered [27]. Zhu et al. [61] studied the influence of liquid absorption and swelling on the permeability of a bidirectional woven jute fabric with two test fluids, motor oil, and water-diluted corn syrup. The tests were performed under a constant flow rate while recording flow front positions with a video camera. Contrary to motor oil, when corn syrup was adopted, liquid absorption and swelling occurred, determining the reduction of porosity and, consequently, permeability.

**Figure 6:**
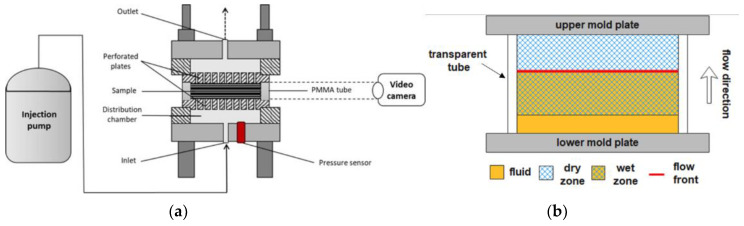
(**a**) Experimental set-up for concurrent out-of-plane unsaturated permeability and compaction characterization and (**b**) an illustration of transverse flow front advancement and fiber-bed compaction ([62] Copyright (2021) with permission from Elsevier).

Although it is quite difficult to apply this method for the measurement of the out-of-plane permeability due to the short distance involved, Kabachi et al. [62] made use of this method to characterize, simultaneously, the unsaturated out-of-plane permeability and the compaction of two glass fabrics: a twill 2/2 woven and a biaxial ±45° non-crimp fabric. Permeability decreased when the degree of compaction and, consequently, the fiber volume fraction increased, as expected. Colored silicon oil was injected under a constant flow rate of 1.5 cm^3^/s. The set-up, together with flow front advancement and fabric compaction, is illustrated in Figure 6.

Almazán-Lázaro et al. [63] applied computer vision to control in-plane flow front velocity to keep a constant optimum value required to achieve improved final mechanical properties by reducing void formation. A video camera was used to monitor the flow front while its velocity was measured and compared to the optimum value, with a controller system adjusting the flow through an appropriate valve.

In many cases, visual monitoring is used to validate other measurement techniques [64,65,66,67,68,69,70]. Its main limitation is the impossibility of observing flow inside the composite structure, especially when a large sample is considered; however, it remains the simplest and most cost-effective method for permeability measurement.

### 3.3. Methods Based on Ultrasonic Wave Propagation

Ultrasonic sensors are generally used for non-destructive evaluation or, more recently, for structural health monitoring [71]. Similarly, they allow non-invasively measuring of both the in-plane and the out-of-plane permeability (embedded sensors or contact between probes and samples are not required). Moreover, resin flow can be monitored without transparent molds and also visualized through opaque reinforcement materials [72]. However, the major limit is still the low thickness of the used preform due to the limited resolution depth of ultrasound in the MHz range.

There are different methods based on the propagation of ultrasonic waves. In this section, they are divided into two categories, methods based on longitudinal waves and methods based on Lamb waves.

#### 3.3.1. Ultrasonic Methods Based on Longitudinal Waves

**Figure 7:**
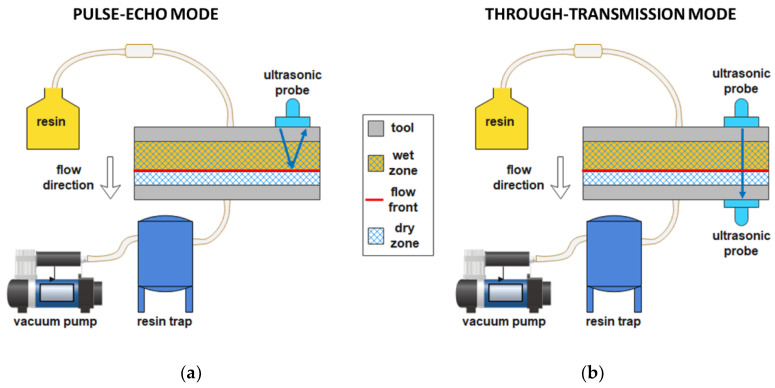
Experimental set-up for out-of-plane permeability measurement by ultrasonic wave propagation with (**a**) pulse-echo mode or (**b**) through-transmission mode.

Longitudinal waves are waves characterized by the same direction of particle motion and wave propagation. Two different methods are based on longitudinal waves, the pulse-echo mode, where a single probe works simultaneously as emitter and receiver, and the through-transmission mode, where two probes are needed (Figure 7) [73]. Lionetto et al. [52] estimated the out-of-plane permeability of three carbon fiber preforms by using the pulse-echo mode. The ultrasonic waves generated by the probe were reflected at the air-fluid interface and returned to the probe. The distance between the flow front and the transducer was calculated from the time-of-flight Δt (s) of the reflected echo:

(8) $$x_{f}=\frac{v\Delta t}{2}$$

where v (m/s) is the longitudinal wave velocity. The term 2 is present in Equation (8) only in the set-up configuration in a pulse-echo mode where the ultrasonic wave travels from the transducer to the preform and back from the preform to the transducer. Then, permeability was determined from Equation (7) by extrapolating the slope of the linear fitting of x_f_ versus the square root of the infusion time. The unsaturated permeability values were lower than the saturated values measured by the gravimetric method. The balanced preform showed the highest permeability, while the unstitched unidirectional preform showed the lowest values.

The set-ups in through-transmission mode are the evolution of that one reported by Stöven et al. [74], used to study the unsaturated out-of-plane permeability of a multiaxial carbon non-crimp fabric and a biaxial glass non-crimp fabric. Recently, Konstantopoulos et al. [75] used the through-transmission mode to study the out-of-plane unsaturated permeability of three preforms, a biaxial ±45° carbon non-crimp fabric, a carbon plain weave, and a glass twill weave 2/2. The ultrasonic waves propagated from the emitter to the receiver probe through the thickness of the preform. The time-of-flight is shortened during preform impregnation as sound travels faster within a saturated medium. For all preforms, permeability increased with preform thickness and decreased with fiber volume fraction.

Becker et al.’s [76] benchmarking study demonstrated the reliability and accuracy of the measurements based on ultrasonic wave propagation but also the need to improve the reproducibility of this method.

#### 3.3.2. Ultrasonic Methods Based on Lamb Waves

**Figure 8:**
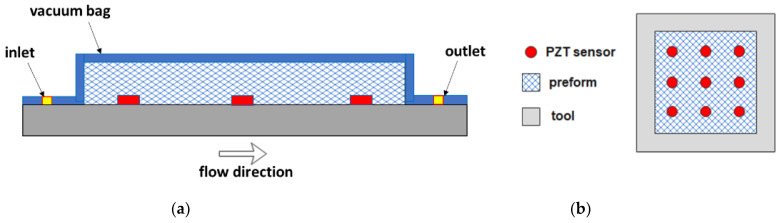
(**a**) Experimental set-up and (**b**) sketched cross-section of PZT sensors-based flow front monitoring (adapted from Liu et al. [78]).

Lamb waves are waves guided along a plate with particle motion lying in the plane containing the direction of wave propagation and the direction perpendicular to the plate. The Leaky Lamb wave phenomenon is induced when an ultrasonic set-up with the emitter and receiver probes angulated on the same sample size (pitch-catch configuration) irradiates a solid plate immersed in fluid. Lamb waves can leak into the liquid through the solid-liquid interface with a consequent energy and amplitude reduction. This property can be used to detect fiber impregnation [77]. This method, compared to the longitudinal waves method, is suitable for thick preforms thanks to the capability of guided waves to propagate over long distances. Liu et al. [78] realized a multi-functional piezoelectric (PZT) sensor network (nine circular PZTs placed on an aluminum plate mold, Figure 8) to study the impregnation process of a unidirectional carbon fiber sample infused with epoxy resin. When the plate got in touch with resin during infusion, Lamb waves were attenuated, and resin arrival was tracked. The embedded configuration of the sensor proved to be effective for flow monitoring and also for the entire life-cycle health condition monitoring [79].

Yu et al. [77] realized a hybrid piezoelectric-fiber sensor network embedded inside a carbon fiber preform to monitor the three-dimensional resin flow front. They used PZTs for flow front monitoring in the thickness direction and Fiber Bragg Grating (FBG) sensors to monitor the flow front inside the sample.

### 3.4. Methods Based on Dielectric Sensors

The methods based on dielectric sensors rely on the dielectric properties of reinforcement and fluid. They include capacitive and resistive sensors used for detecting the resin arrival.

#### 3.4.1. Methods Based on Capacitive Sensors

**Figure 9:**
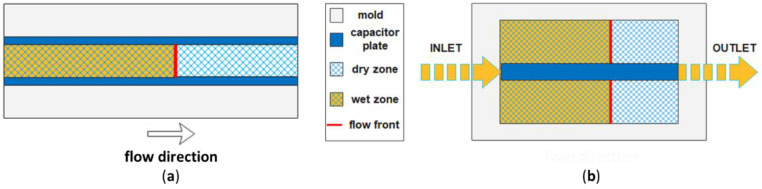
(**a**) Experimental set-up for flow front monitoring with a parallel-plate capacitor and (**b**) sketch of in-plane flow front advancement (adapted from Qi et al. [80]).

Capacitive sensors are based on the change in capacitance of the reinforcement when it is impregnated with the liquid resin. Qi et al. [80] measured the unsaturated in-plane permeability of two glass fiber preforms. They used an epoxy resin as a test fluid, a transparent polymethyl methacrylate (PMMA) mold to visualize the resin flow front, and a parallel-plate capacitor composed of two copper foil tapes 1.5 cm wide and of the same length of the mold (Figure 9a). During infusion, two biphasic regions are present, a wet and a dry zone (Figure 9b), both contributing to the total capacitance C (F) between the two armatures of the capacitor, given by the following equation:

(9) $${C=C}_{\mathrm{wet}}{+C}_{\mathrm{dry}}{=\varepsilon \varepsilon }_{0}\frac{A}{d}$$

where A (m^2^) is the area of armatures and d (m) is the distance between them, ε (-) is the dielectric constant, and ε_0_ (F/m) is the vacuum permittivity. The dielectric constant of a region formed by two different materials α and β, in this case, fibers and resin (wet zone) or fibers and air (dry zone), is given by Lichtenecker’s equation [81]:

(10) $${\log(\varepsilon )=V}_{\alpha }\log\left(\varepsilon_{\alpha }\right){+V}_{\beta }\log\left(\varepsilon_{\beta }\right)$$

where V_α_ and V_β_ (-) and ε_α_ and ε_β_ (-) are the volume fractions and the dielectric constants of the two materials. The volume fraction and the dielectric constant were the only two unknown parameters in Equation (9), obtained by recording the flow front position x_f_ and plotting C as a function of x_f_. After the described calibration procedure, C was used to monitor the front position, and the unsaturated in-plane permeability was derived from Equation (7).

Rubino et al. [65] employed three dielectric sensors to monitor resin flow and to compare resin infusion with and without resin preheating by a microwave heating system. The analyzed sample consisted of twelve layers of glass twill 2/2 fabric infused with epoxy resin, while the dielectric sensors were formed by two square copper plates (25 × 25 mm^2^). The impregnation of the preform was definitely faster in the case of resin preheating.

A benchmark exercise [82] proved the reproducibility and reliability of unsaturated in-plane permeability measurements using capacitive sensors. Dielectric capacitive sensors are cheap and appropriate for thick preforms but not for conductive fibers (preform and sensor must be separated through a non-conductive material). In addition, constant distance and parallelism between armatures are required. Pouchias et al. [69] designed a flexible dielectric sensor suitable for complex shape molds. This sensor consisted of two co-planar rectangular electrodes placed on the surface of the preform for flow front monitoring and a ground-backplane to reduce the effects of parasitic capacitances, all coated with a dielectric polyimide layer to prevent contact of the sensor with other conductive elements. The capability of the designed sensor to measure resin flow was confirmed by comparing it with visual monitoring during a vacuum infusion process with a carbon fiber preform and an epoxy resin as the testing materials.

#### 3.4.2. Methods Based on Resistive Sensors

Sánchez del Río et al. [67] implemented a simple, flexible, and non-invasive application of resistive sensors, where two parallel carbon fiber yarns were printed with a 3D composite printer on a nylon peel-ply and used as sensors for resin flow front monitoring. The two yarns were printed at a distance of 1 mm and embedded in a nylon matrix except for the tip. The testing material included an epoxy vinyl ester resin, an E-glass plain woven fabric, and a metallic mold. The voltage changed due to the resistance change detected by the sensor when resin wetted the tip of carbon fiber yarns, and the in-plane permeability was calculated from Equation (7) by plotting the resin flow front position versus the time of resin arrival. The reliability of these sensors, which were placed perpendicular to the flow direction, was confirmed by comparing the results with those obtained from visual monitoring. Sensors were more accurate in detecting the resin arrival and were also placed among the plies along the thickness direction to assess the resin front velocity on each layer.

Examples of carbon nanomaterials successfully used as sensors for resin flow monitoring are carbon nanotubes (CNTs) coated sensors [66,83,84] and graphene-coated sensors [85,86], of which the former proved to be better performing [87]. Dai et al. [66], in particular, realized a 400 μm thick areal sensor formed by a resistive CNTs network deposited on an E-glass fabric mat using a dip-coating process. Both linear and radial flow experiments were carried out under a vacuum with an epoxy resin as a test fluid. During linear flow experiments, a preform consisting of six unidirectional E-glass plies was tested, and two sensors were adopted to monitor the flow front position on the top and bottom of the preform, which was at the same time recorded with a video camera. In this way, the validation of sensors was obtained. Flow front monitoring was based on the decrease in the electrical conductivity of the sensor at the passage of resin because of the reduction of contacts between the CNTs. The radial flow experiments, instead, proved the spatial flow mapping capability of the sensor with the integration of the electrical impedance tomography (EIT) approach. In this case, a preform of six plain weave E-glass plies was infused with the epoxy resin, and a CNT-based fabric sensing layer and a video camera were used for EIT measurements and visual monitoring, respectively. Carbon nanotube-coated sensors have proved to be suitable not only for flow monitoring but also for the entire fabrication process and the following structural health monitoring [88].

Tifkitsis et al. [89] designed a lineal sensor composed of two twisted copper wires (diameter of 127 μm and pitch of 500 twists/m) with an insulating polyurethane enamel coating. The resin flow front position x_f_ during the infusion process of a preform consisting of nine layers of a 5H satin weave carbon fabric was determined from the admittance Y values measured by the sensor:

(11) $$x_{f}=\frac{{Y-Y}_{d}}{Y_{w}{-Y}_{d}}L$$

where Y_w_ and Y_d_ (S) are the admittance of the fully wetted and dry sensor, respectively, and L (m) is the sensor length, and results were compared with visual monitoring. This sensor was applied to acquire the flow monitoring data required for a real-time probabilistic estimation of process outcomes of the RTM fabrication process of a carbon fiber-reinforced composite flat part with a recessed edge [90].

### 3.5. Methods Based on Fiber Optic Sensors

**Figure 10:**
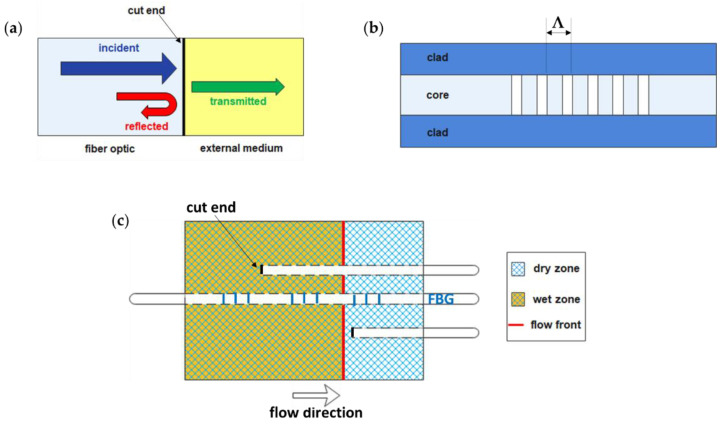
(**a**) Sensing principle of fiber optics based on Fresnel law (adapted from Chehura et al. [96]), (**b**) a sketch of the Fiber Bragg Grating structure, and (**c**) an example of embedded fiber optics application for flow front monitoring.

Fiber optics are among the first sensors to be used for flow front monitoring by detecting differences in optical or mechanical properties [91,92,93]. Flow front monitoring can be realized by cutting fiber optics from one side. In this way, because of the interaction between the optical radiation and the external medium (air or resin), a change in the reflection coefficient R (-) occurs (Figure 10a), which can be calculated from Fresnel law [94]:

(12) $$R={\left(\frac{n_{f}{-n}_{m}}{n_{f}{+n}_{m}}\right)}^{2}$$

where n_f_ (−) is the refractive index of the fiber optic, and n_m_ (−) is the refractive index of the external medium. In Figure 10b, the structure of a Fiber Bragg Grating (FBG) sensor is shown, which is a fiber optic characterized by a short core section with the refractive index modulated according to a grating period Λ. Mechanical (Δε (με)) or thermal (ΔT (°C)) stress induces a shift of the reflected wavelength (Bragg wavelength) Δλ_B_ (pm) given by [95]:

(13) $$\Delta \lambda_{B}{=S}_{\varepsilon }{\Delta \varepsilon +S}_{T}\Delta T$$

where S_ε_ (pm/με) and S_T_ (pm/°C) are the strain and temperature sensitivity, respectively. A typical application of fiber optics is illustrated in Figure 10c.

Marrazzo et al. [97] selected fiber optics to monitor resin flow front and temperature during infusion within a carbon fiber panel. While fiber optics with cut ends were used for flow front monitoring, FBG sensors with a Bragg grating length of 10 mm were used for temperature monitoring [57]. Yu et al. [77], instead, used FBG sensors in their hybrid piezoelectric-fiber sensor network described in Section 3.3.2 to monitor resin flow front in the thickness direction through the shift of Bragg wavelength caused by contact between resin and sensors. Chehura et al. [96] applied embedded fiber optics to monitor the filling stage and the cure cycle of a carbon fiber-reinforced tail cone, as the refractive index of resin is also sensitive to density variation [98].

Jeong et al. [64] did not use standard FBG sensors but long-gauge FBG sensors (gauge length 100 mm) to realize a distributed optical frequency domain reflectometry (OFDR) sensing system with a spatial resolution of less than 1 mm. The long-gauge FGB sensor was placed in the middle of the preform thickness to measure its strain change. The preform was made up of 20 plies of a plain weave glass fiber fabric infused with silicon oil. The flow front position matched the point at which the strain rate was zero, while its value was positive in the impregnated region and negative in the dry region. So determined flow front positions were confirmed by images taken with a camera.

Fiber optics advantages include immunity to electromagnetic interference, the possibility to install many sensors on the same optical fiber, the lack of any interference with carbon fibers as in electrical-based systems, and their market availability. However, potential intrusiveness and signal loss due to fiber bending are their main drawbacks.

### 3.6. Numerical Methods for Permeability Prediction Based on X-ray Microtomography

**Figure 11:**
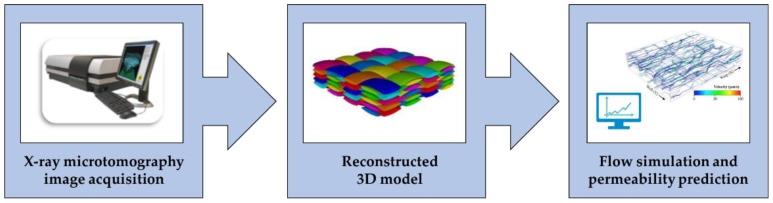
Illustration of the procedure for permeability prediction using models generated from 3D X-ray microtomography images (adapted from Yousaf et al. [107] and Ali et al. [108]).

Numerical permeability prediction is generally based on geometric models of the reinforcements [99,100,101,102,103], which, however, do not represent their actual complex microstructure, including several heterogeneities and defects. Numerical methods for permeability prediction use models generated from 3D X-ray microtomography images of the dry reinforcement, as shown in Figure 11. X-ray microtomography (micro-CT) is a radiographic imaging technique commonly used for the characterization of polymer composites [24,104,105,106].

After tomographic image acquisition by micro-CT, a 3D model can be reconstructed, which represents the realistic domain for resin flow simulation, and permeability is predicted by numerically solving the governing equations of fluid dynamics.

Zeng et al. [109] first exploited 3D X-ray microtomography images for CFD analysis to simulate resin infusion within the real structure of samples. Aziz et al. [50] predicted the out-of-plane permeability of dry carbon fiber preforms manufactured by automated fiber placement through flow simulations on geometrical models reconstructed from computed tomographic images, and results were validated using the experimental data obtained with the flow rate measurement method. Similarly, Caglar et al. [54] investigated the effects of spherical inclusions on the in-plane saturated permeability of a 2 × 2 twill weave glass fabric using the gravimetric method for validation.

Ali et al. [23,108,110] predicted the in-plane and out-of-plane permeability of a 3D orthogonal and a 3D angle interlock carbon fabric at different values of fiber volume fraction from tomographic images obtained during compression tests. The results agreed with the experimental data. Similarly, Yousaf et al. [107] studied the effects of nesting on the permeability of an E-glass plain woven fabric. Ghafour et al. [111] applied this method in the case of plant-based reinforcement materials since it is difficult to assess how their complex microstructure changes during compression. The microstructure of two flax fiber mats, the first one made of flax and polypropylene (10 wt%) fibers and the second one made of only flax fibers, was analyzed during out-of-plane compression tests. The pore and fiber volume fractions, mean pore and fiber diameters, specific surface area, and directional tortuosities were estimated, while the components of the permeability tensor were calculated by numerical simulations.

Although this is not an experimental method for permeability measurement, it can be considered a virtual testing approach representing a viable alternative to experimental procedures. The interest in image-based permeability prediction has increased in recent years, as demonstrated by a new benchmark exercise, which also aims to develop guidelines for numerical permeability prediction. In fact, despite this method having many advantages, such as the possibility to consider the material variability and small-scale parameters, study multiple influencing factors, and reduce the material waste, some challenging issues include the need for elevated computing power, the potential errors related to image acquisition and geometry reconstruction, the presence of several numerical methods and influencing parameters, and the multi-scale porosity of fiber reinforcements [112].

## 4. Conclusions and Future Perspectives

The measurement of preforms permeability in liquid composite molding is of paramount importance to optimize the resin infusion process. Currently, however, there are no standards for making such measurements. Over the years, many methods have been introduced to determine permeability in the in-plane and out-of-plane directions and under saturated or unsaturated conditions. These methods differ mainly in the way the resin flow front is monitored during the reinforcement impregnation.

The aim of this work was to analyze the most commonly used and newest techniques of the last six years, with their pros and cons. Numerous sensors have been used over the years, starting from fiber optics to piezoelectric and carbon nanomaterials. In addition to those mentioned in this overview, other sensors, mostly used in the past, include thermocouples [98,113], point- and lineal-voltage sensors [114], and pressure sensors [68,115,116]. Undoubtedly, the video camera recording technique remains among the simplest, cheapest, and most effective methods. In the future, however, non-invasive nanosensors could be preferred since they are useful not only for monitoring the injection and cure processes but, thanks to the possibility of integrating them inside the composite material, also for structural health monitoring. Non-negligible support in improving the procedures adopted for permeability measurement is certainly provided by computational fluid dynamics (CFD).

## Figures and Tables

**Table 1:** Experimental methods for permeability measurement.

Method	Measured Permeability	Advantages	Drawbacks
Flow Rate Measurement	saturated; in-plane and out-of-plane	simplicity, low cost, suitable for all materials, non-destructiveness (gaseous test fluid)	fluid compressibility for gaseous test fluid
Video Recording	unsaturated; in-plane and out-of-plane	simplicity (in-plane), low cost, suitable for all materials	only for superficial monitoring, transparent mold required
Ultrasonic Wave Propagation	unsaturated; in-plane and out-of-plane	non-invasiveness, low cost, suitable for all materials	complexity, limited resolution (longitudinal waves)
Dielectric Sensors	unsaturated; in-plane and out-of-plane	low cost, non-invasiveness, integrability (resistive nanosensors)	not suitable for conductive materials (separating layer required)
Fiber Optic Sensors	unsaturated; in-plane and out-of-plane	immunity to electromagnetic interference	invasiveness, signal loss due to fiber bending

## Data Availability

Data available on request.
